# Supplementary material for: Waning-and-waxing shape changes in ionic nanoplates upon cation exchange
Source: Nat Commun. 2024 Jun 8;15:4899. doi: 10.1038/s41467-024-49294-x (PMC11162454; doi:10.1038/s41467-024-49294-x)
Supplement: Supplementary file 1 — Supplementary Information [file 41467_2024_49294_MOESM1_ESM.pdf]

Supplementary Information for

Waning-and-waxing shape changes in ionic nanoplates upon cation exchange

Zhanzhao Li[1], Masaki Saruyama[1*], Toru Asaka[2] and Toshiharu Teranishi[1*]

[1]Institute for Chemical Research, Kyoto University, Gokasho, Uji, Kyoto 611-0011, Japan.

[2]Division of Advanced Ceramics, Nagoya Institute of Technology, Nagoya, Aichi 466-8555, Japan.

*e-mail: saruyama@scl.kyoto-u.ac.jp; teranisi@scl.kyoto-u.ac.jp

Contents:

Supplementary Figs. 1–22

Supplementary Table 1

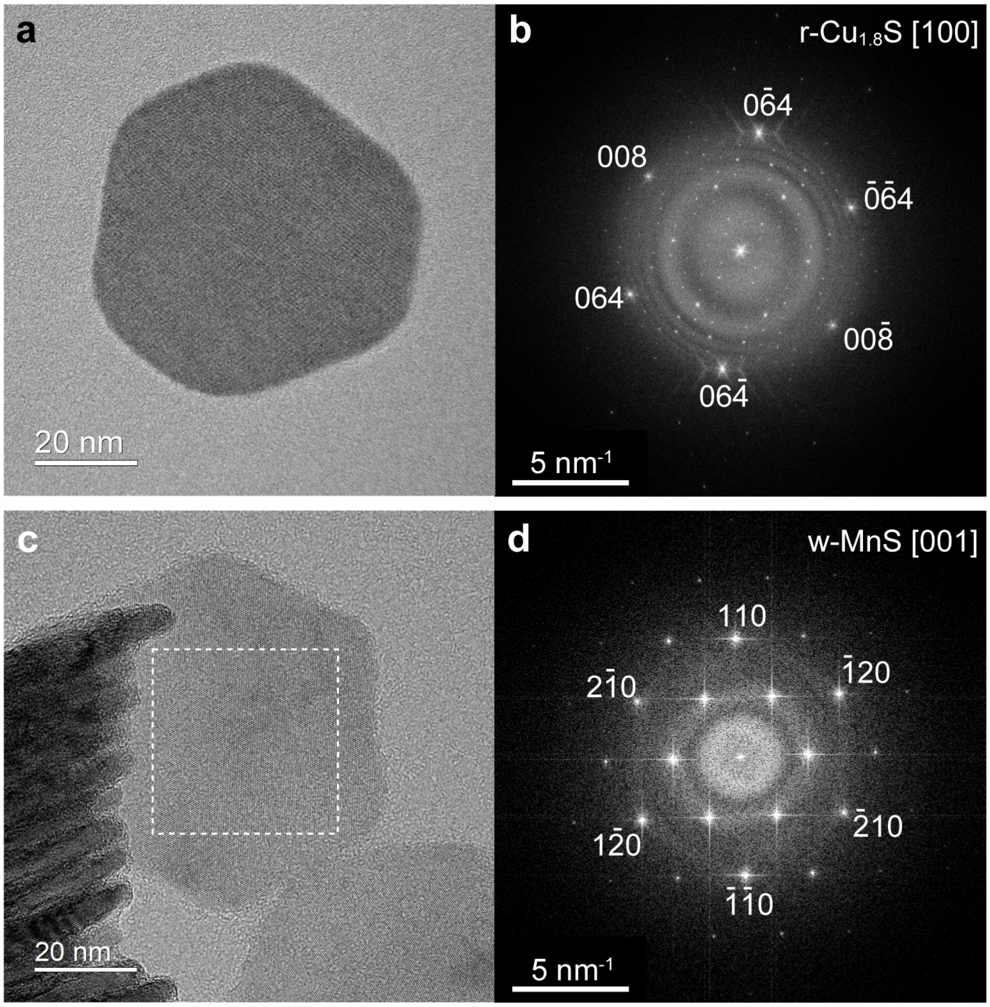

Supplementary Fig. 1 HRTEM observation of $Cu_{1.8}S$ and MnS NPLs. (a) HRTEM image and (b) FFT pattern of host $Cu_{1.8}S$ NPLs. (c) HRTEM image and (d) selected area FFT pattern of MnS NPLs.

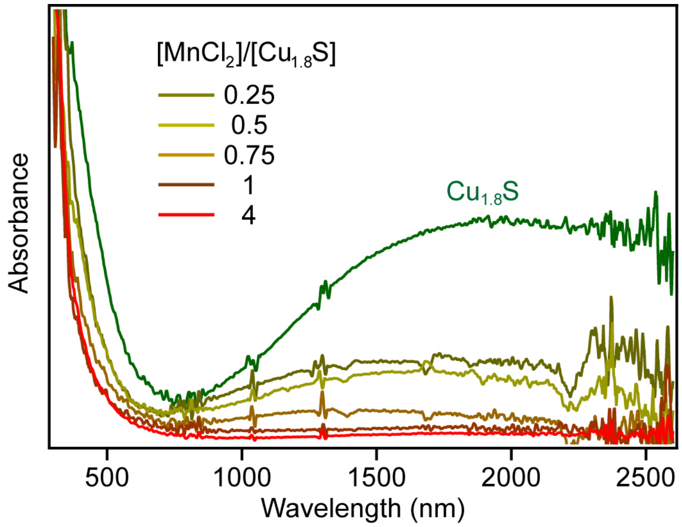

Supplementary Fig. 2 Optical absorption of $Cu_{1.8}S$ NPLs and the CE products. UV–vis–NIR spectra of $Cu_{1.8}S$ NPLs and Mn^{2+} CE products generated using various $[MnCl_2]/[Cu_{1.8}S]$.

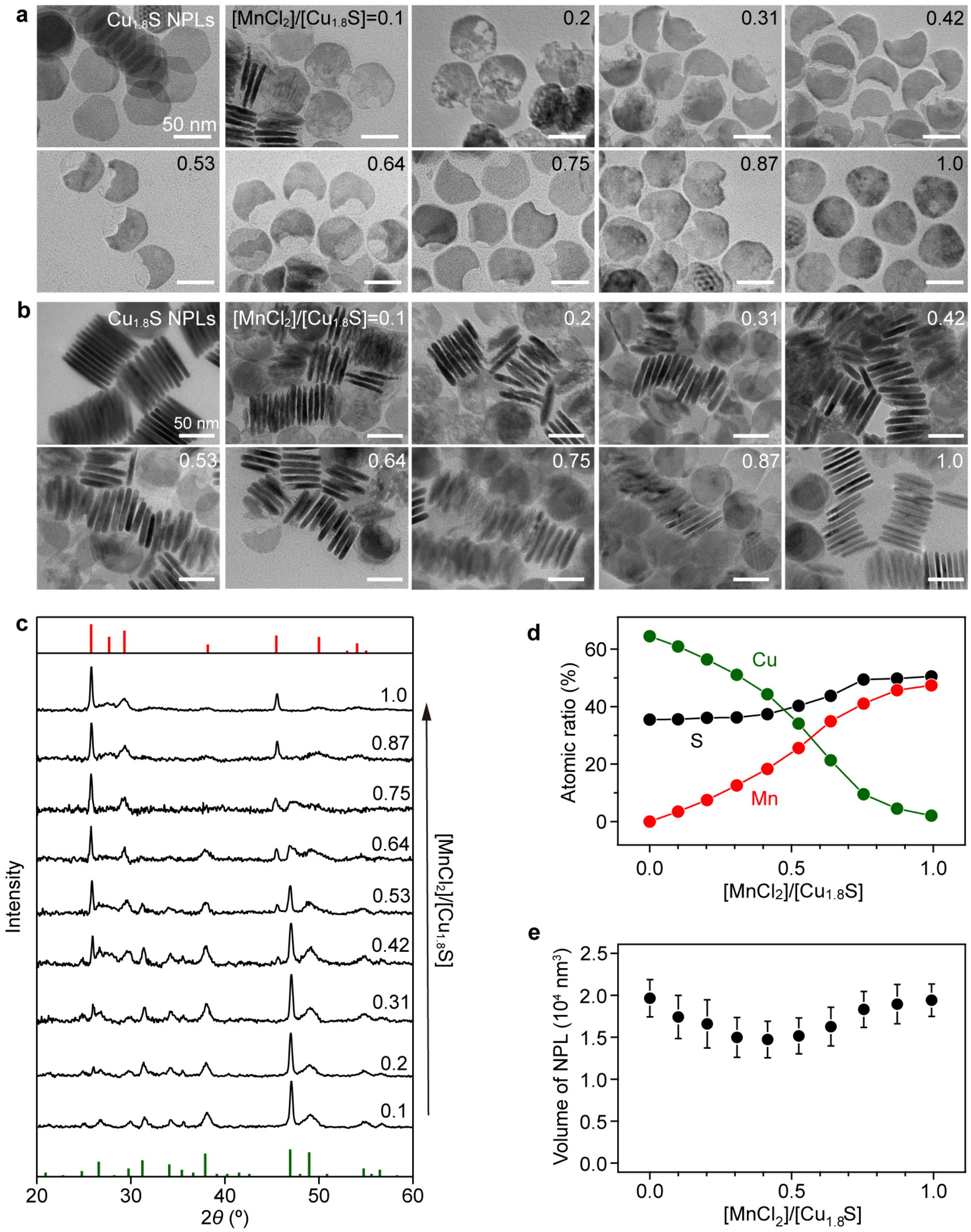

Supplementary Fig. 3 Successive Mn^{2+} CE of $Cu_{1.8}S$ NPLs. (a,b) TEM images, (c) XRD patterns and (d) compositional changes of $Cu_{1.8}S$ NPLs and $Cu_{1.8}S$–MnS HNPLs formed by successive CE reactions with Mn^{2+}. (a) Top views and (b) side views. Scale bars = 50 nm. (e) Volumes of individual $Cu_{1.8}S$–MnS HNPLs at each CE step. Error bars represent standard deviations calculated from standard deviations of basal areas (n = 10–15) and thicknesses (n = 18–22). r: roxbyite; w: wurtzite. Reference XRD patterns: w-MnS (red, ICDD-PDF 01-089-4089) and r-$Cu_{1.8}S$ (black, ICDD-PDF 00-064-0278).

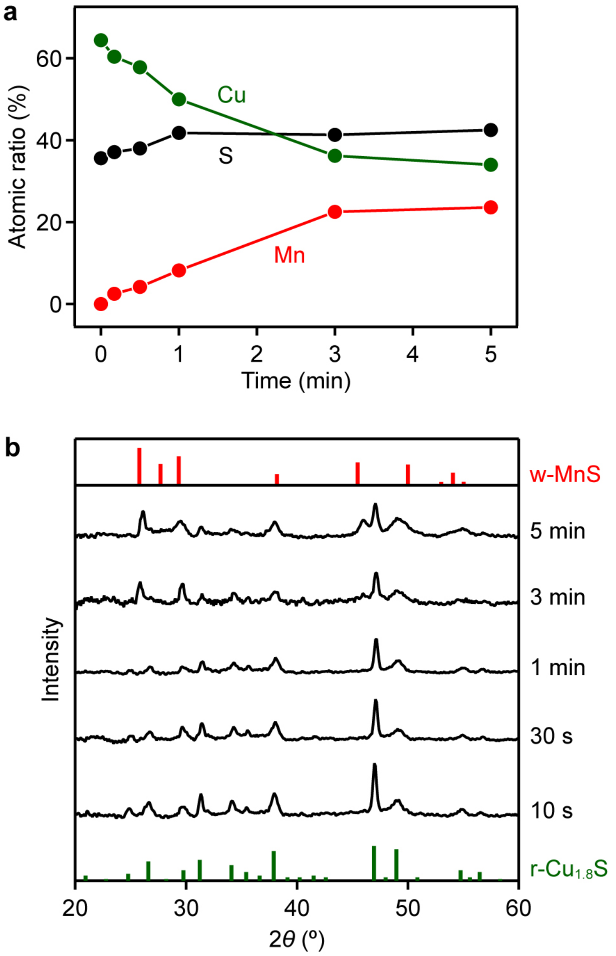

Supplementary Fig. 4 Composition and crystal structure changes during the waning process. Temporal change of (a) EDX results and (b) XRD patterns of the products obtained during partial CE. Reaction conditions: $[MnCl_2]/[Cu_{1.8}S] = 0.5$. Reference XRD patterns: w-MnS (ICDD-PDF 01-089-4089) and r-$Cu_{1.8}S$ (ICDD-PDF 00-064-0278).

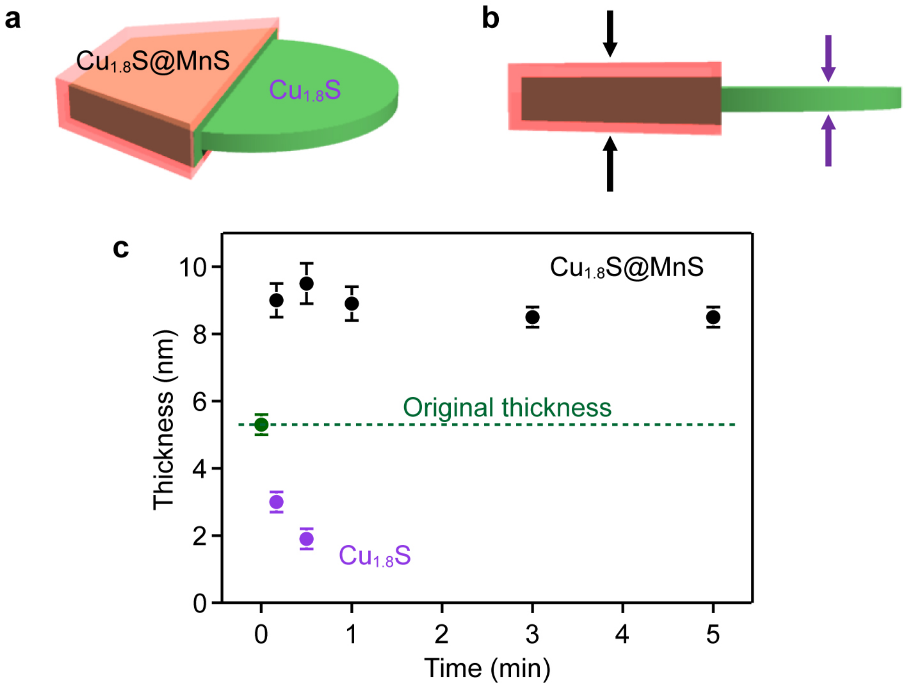

Supplementary Fig. 5 Thickness evolution during the waning process. (a,b) Structure model of $Cu_{1.8}S$-MnS HNPLs in the early stage of partial CE and (c) temporal change of NPL thickness during partial CE. Error bars represent standard deviations. $Cu_{1.8}S$ part: n = 50 for 0 min, n = 10 for 0.17 min and n = 10 for 0.5 min. $Cu_{1.8}S$-MnS part: n = 10 for 0.17 min, n = 10 for 0.5 min, n = 20 for 1 min, n = 20 for 3 min and n = 50 for 5 min. Reaction conditions: $[MnCl_2]/[Cu_{1.8}S]$ = 0.5. The thin naked $Cu_{1.8}S$ part disappeared after 1 min.

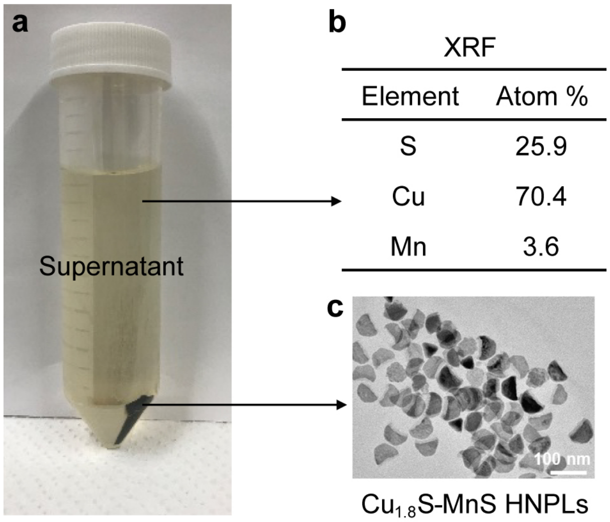

Supplementary Fig. 6 Composition analysis of the supernatant in purification step after waning process. (a) Photo of centrifuged solution in the purification step after partial CE of $Cu_{1.8}S$ NPLs with Mn^{2+} (5 min). (b) XRF results for the concentrated supernatant and (c) TEM image of the precipitated products. Reaction condition: $[MnCl_2]/[Cu_{1.8}S] = 0.5$.

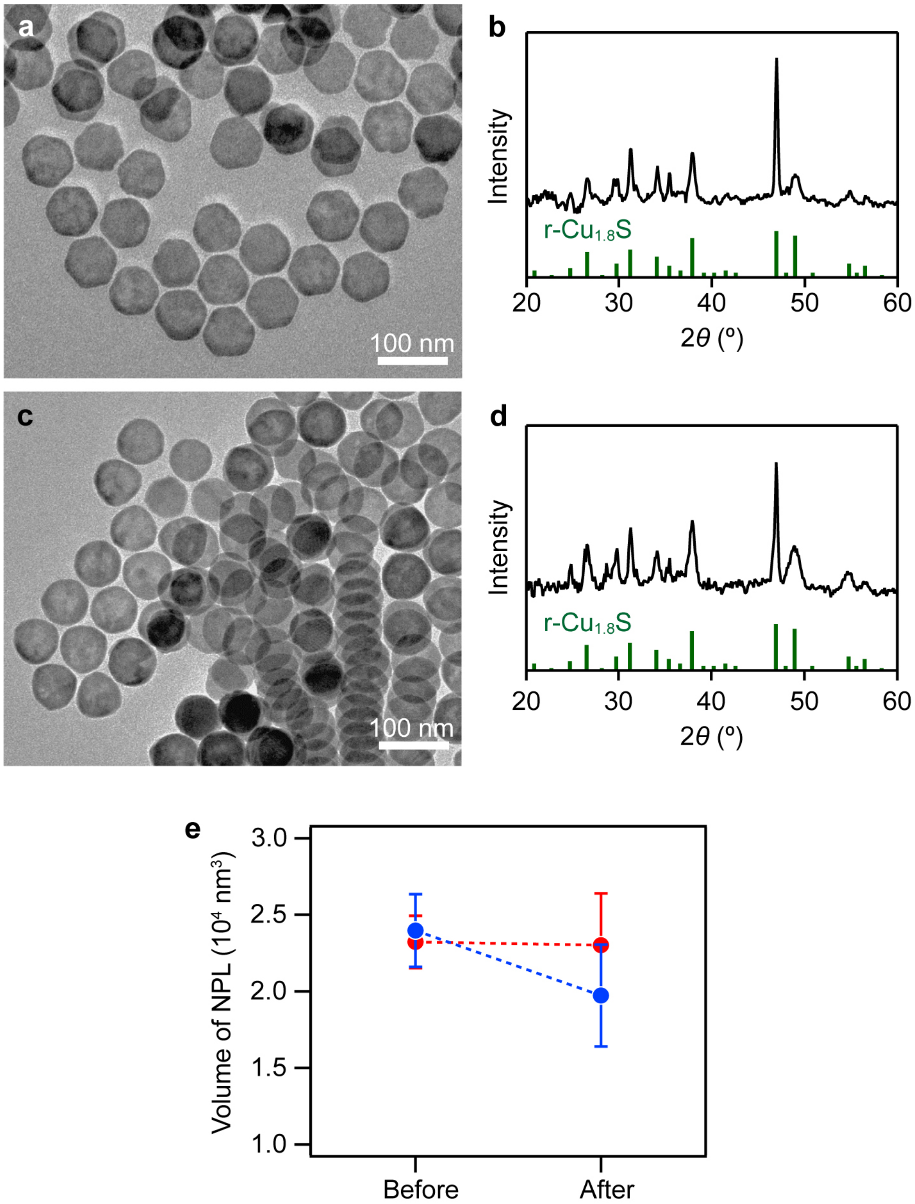

Supplementary Fig. 7 Control experiments without Mn precursor. (a,c) TEM images and (b,d) XRD patterns of the products obtained in control experiments (a,b) without $MnCl_2$ and (c,d) with TOA chloride instead of $MnCl_2$. (e) Volume change of individual $Cu_{1.8}S$ NPLs before and after the control reaction (red) without $MnCl_2$ and (blue) with TOA chloride. Error bars represent standard deviations calculated from standard deviations of basal areas (n = 12–21) and thicknesses (n = 23–29).

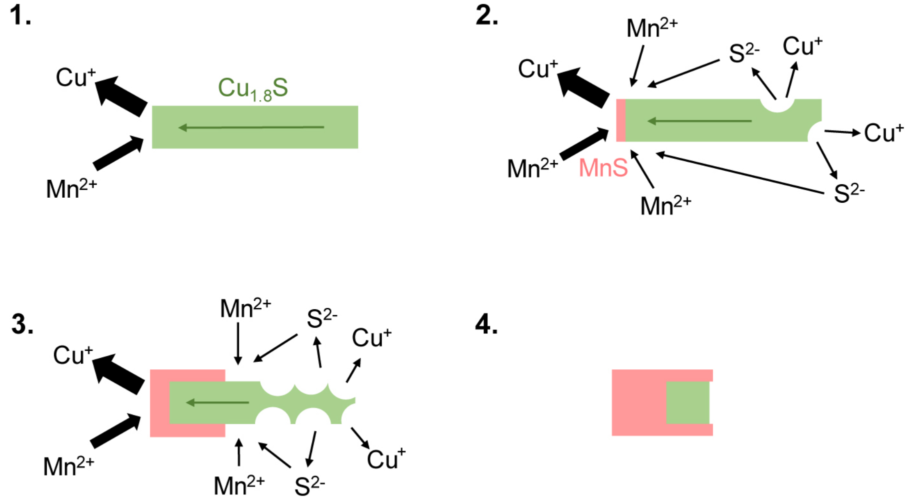

Supplementary Fig. 8 Speculated waning mechanism during the partial Mn^{2+} CE reaction. Schematic of the conversion pathway from $Cu_{1.8}S$ NPL to crescent shaped $Cu_{1.8}S$–MnS HNPL including CE, etching of $Cu_{1.8}S$, and MnS deposition mechanisms from the side view.

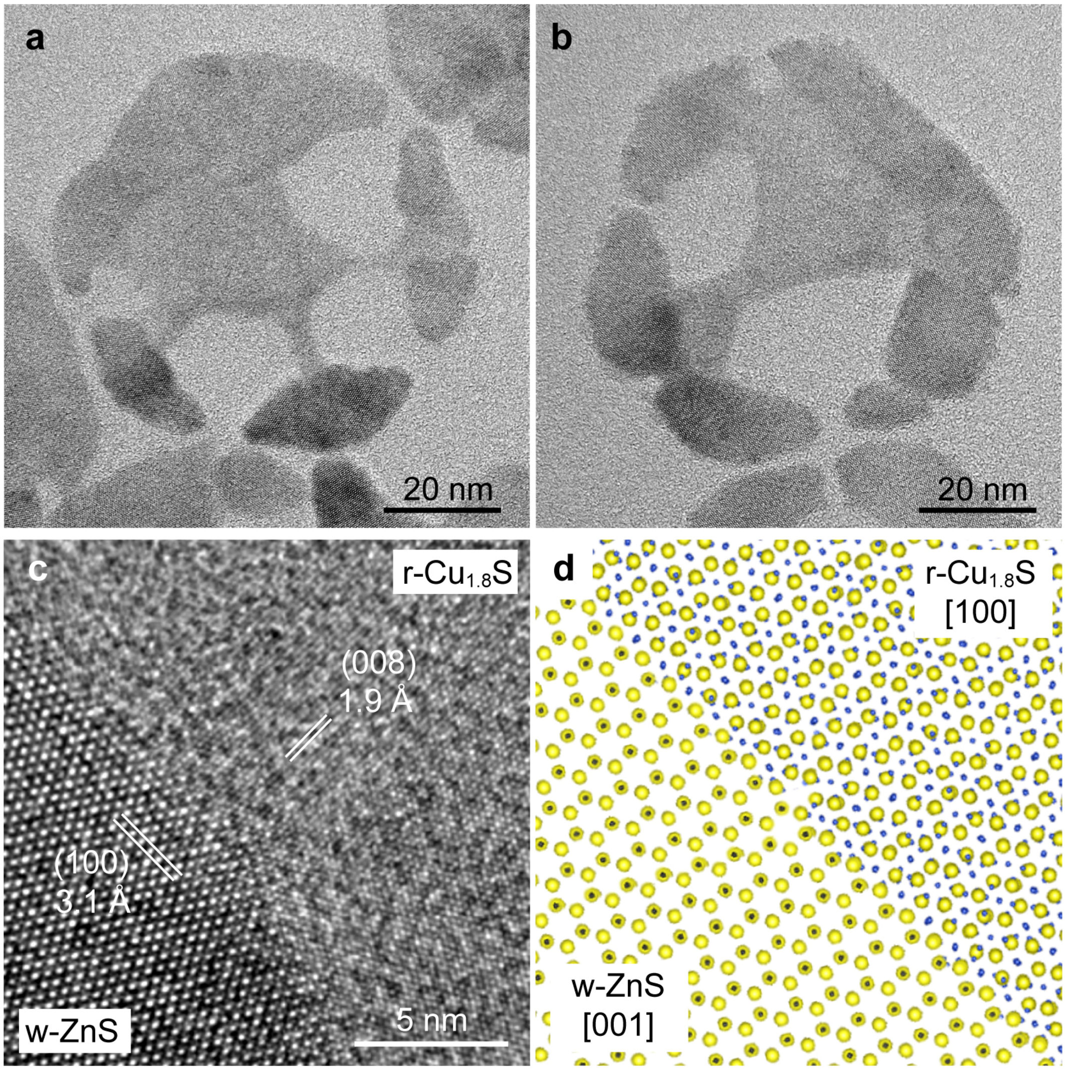

Supplementary Fig. 9 HRTEM observation of chipped $Cu_{1.8}S$–ZnS HNPLs. (a,b) HRTEM images of the chipped $Cu_{1.8}S$–ZnS HNPLs formed by partial CE of $Cu_{1.8}S$ NPLs with Zn^{2+}. Reaction conditions: $[ZnCl_2]/[Cu_{1.8}S] = 0.5$. (c) HRTEM image and (d) structure model of the epitaxial r-$Cu_{1.8}S$/w-ZnS heterointerface.

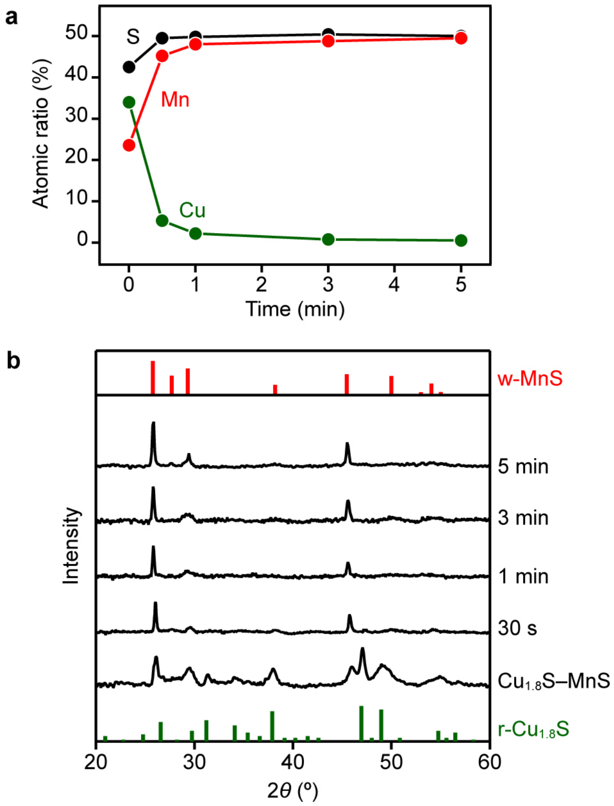

Supplementary Fig. 10 Composition and crystal structure changes during the waxing process. Temporal changes in (a) EDX results and (b) XRD patterns of the products formed by CE of crescent-shaped $Cu_{1.8}S$–MnS HNPLs with Mn^{2+}. Reaction conditions: [$MnCl_2$]/[$Cu_{1.8}S$–MnS] = 4. Reference XRD patterns: w-MnS (ICDD-PDF 01-089-4089) and r-$Cu_{1.8}S$ (ICDD-PDF 00-064-0278).

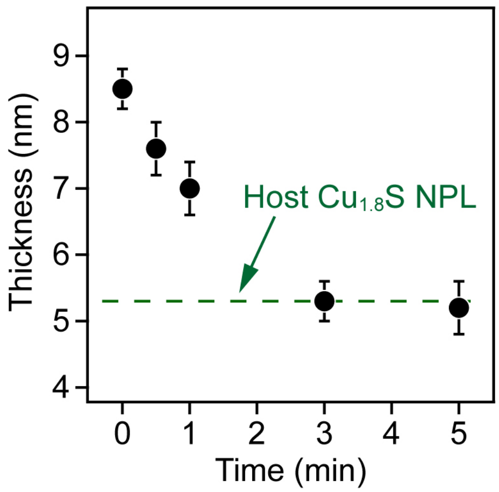

Supplementary Fig. 11 Thickness evolution during the waxing process. Temporal changes in the thickness of individual (H)NPLs in the waxing of crescent-shaped $Cu_{1.8}S$–MnS HNPLs into complete-hexagonal MnS NPLs. Error bars represent standard deviations (n = 10–50).

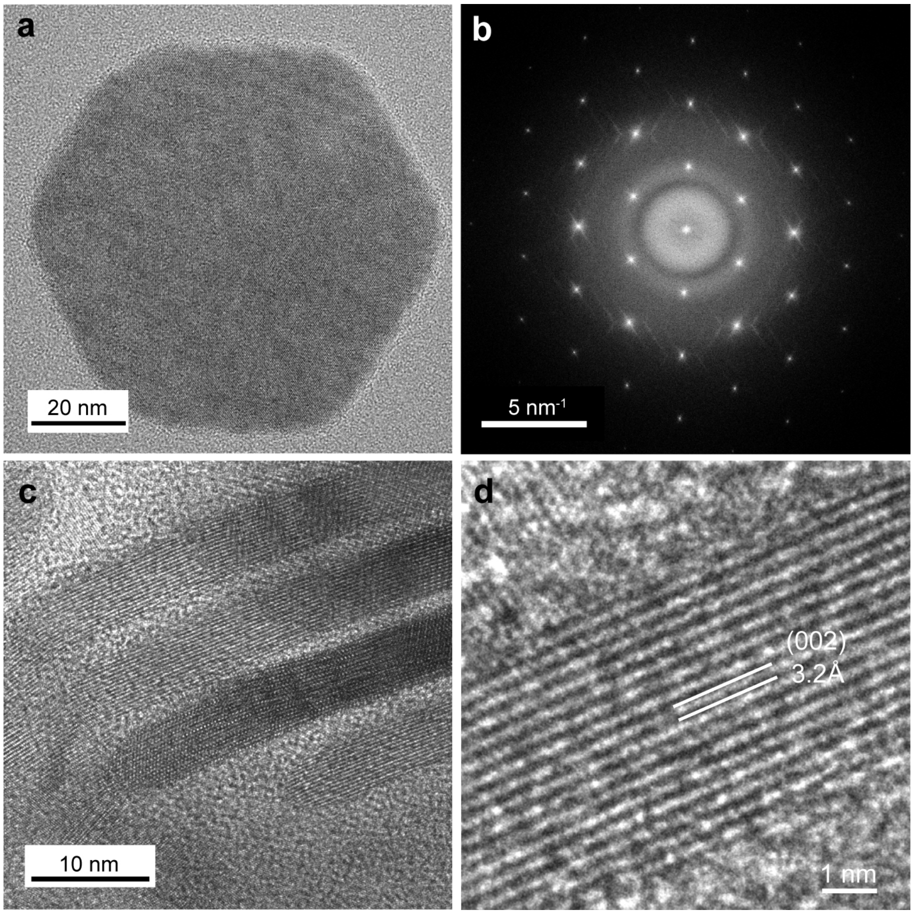

Supplementary Fig. 12 MnS NPLs formed by additional Mn^{2+} CE of the $Cu_{1.8}S$–MnS HNPLs. (a,c,d) HRTEM images and (b) corresponding FFT pattern of hexagonal w-MnS NPLs formed after 3 min CE of $Cu_{1.8}S$–MnS HNPLs with additional Mn^{2+}. (a,b) Top-views and (c,d) side-views. Reaction condition: $[MnCl_2]/[Cu_{1.8}S–MnS] = 4$.

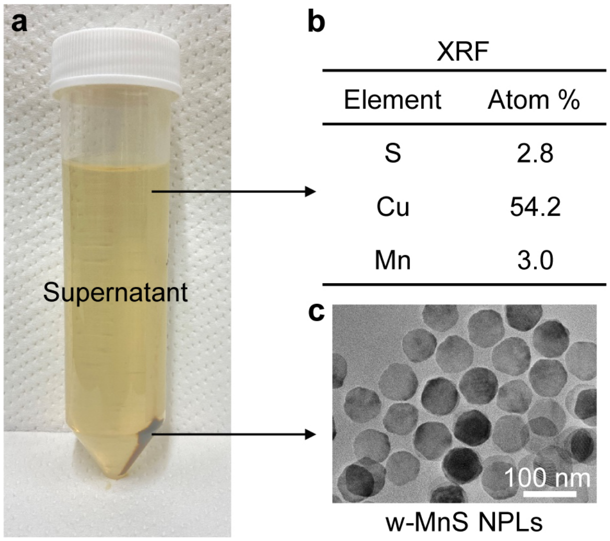

XRF

Element	Atom %
S	2.8
Cu	54.2
Mn	3.0

Supplementary Fig. 13 Composition analysis of the supernatant in purification step after waxing process. (a) Photo of centrifuged solution in the purification step after CE of the crescent-shaped $Cu_{1.8}S$–MnS HNPLs with additional Mn^{2+} at 5 min. (b) XRF results for the concentrated supernatant and (c) a TEM image of the precipitated product. Reaction condition: $[MnCl_2]/[Cu_{1.8}S–MnS] = 4$.

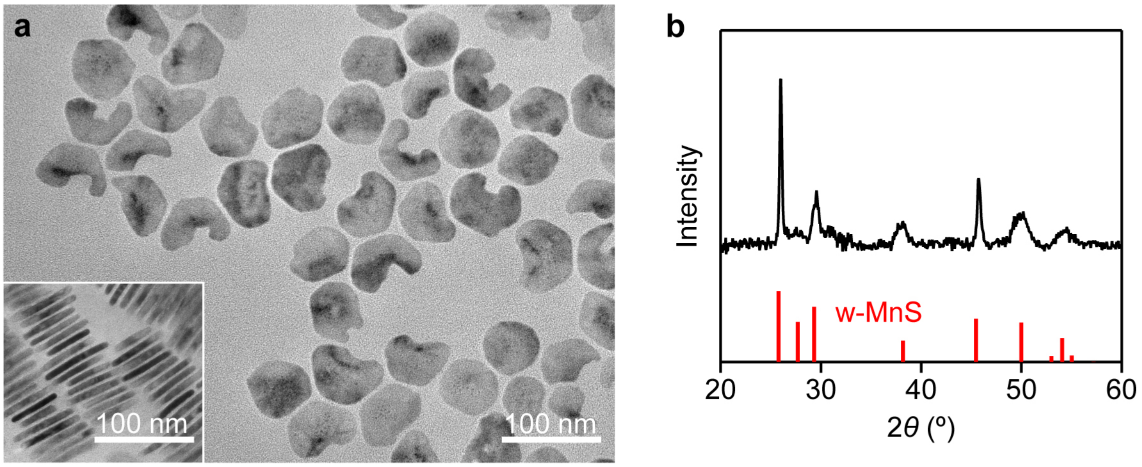

Supplementary Fig. 14 MnS NPLs formed by the additional Mn^{2+} CE of purified $Cu_{1.8}S$–MnS HNPLs. (a) TEM image and (b) XRD pattern of the product formed by the reaction of purified $Cu_{1.8}S$–MnS HNPLs with $MnCl_2$. Inset in (a) shows side-view image. Reference XRD pattern: w-MnS (ICDD-PDF 01-089-4089).

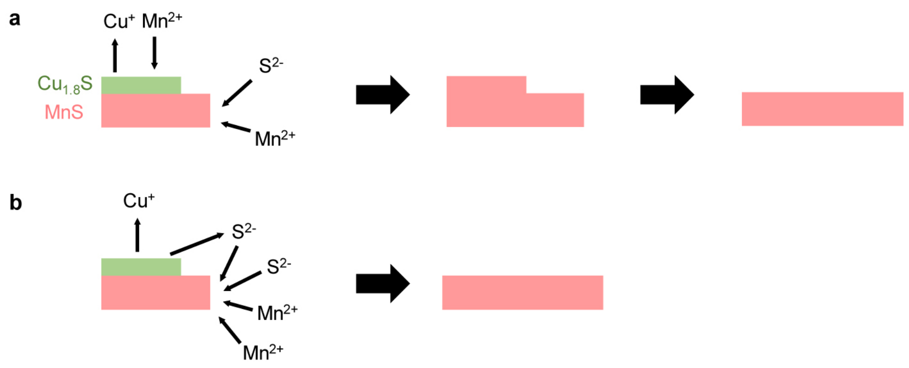

Supplementary Fig. 15 Two possible mechanisms in MnS NPL formation from $Cu_{1.8}S$–MnS HNPL. Schematic of possible conversion pathway of $Cu_{1.8}S$–MnS bilayer structure (at 10 s in waxing process) into MnS NPL from the side view: (a) MnS NPL with uneven thickness after CE of $Cu_{1.8}S$ layer is homogenized to flat, thin MnS NPL; (b) $Cu_{1.8}S$ layer is etched and residual MnS layer is repaired to hexagonal NPL.

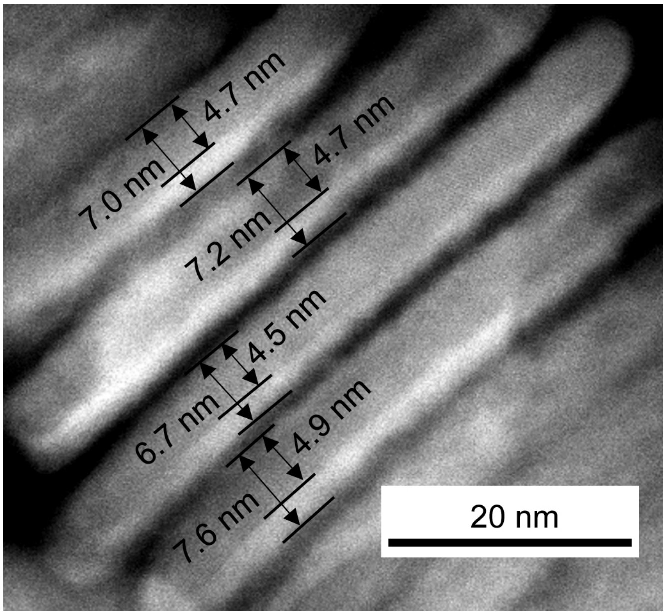

Supplementary Fig. 16 STEM observation of $Cu_{1.8}S$–MnS HNPLs intermediate in waxing process. HAADF-STEM image of $Cu_{1.8}S$–MnS HNPLs at 30 s in the waxing step with information of total thickness and thickness of MnS layer.

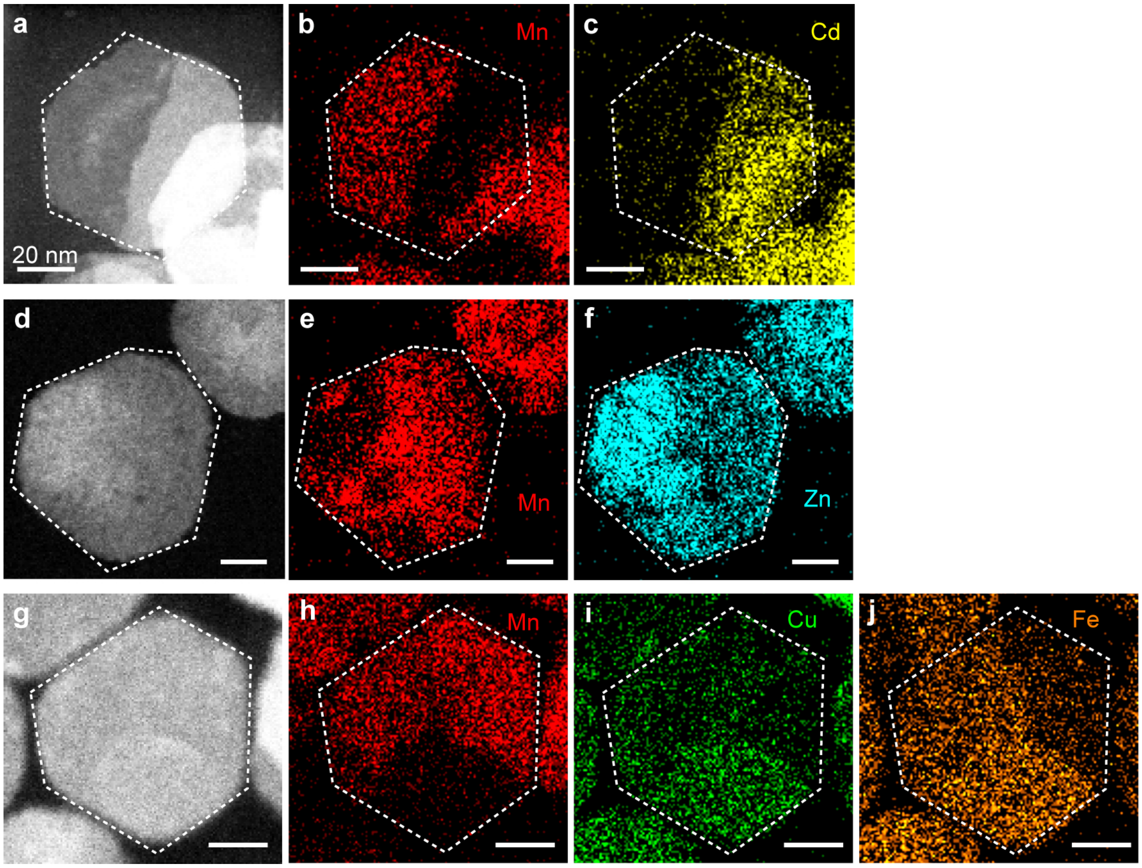

Supplementary Fig. 17 Metal cations distribution in HNPLs after waxing process of $Cu_{1.8}S$–MnS HNPLs. (a,d,g) HAADF-STEM images and (b,c,e,f,h,i,j) STEM–EDX maps of (a–c) MnS–CdS HNPLs, (d–f) MnS–ZnS HNPLs and (g–j) MnS–$CuFeS_2$ HNPLs synthesized by two-step CE. (b,e,h) Red: Mn-K, (c) yellow: Cd-K, (f) blue: Zn-K and (j) orange: Fe-K. Dotted lines outline an HNPL. Scale bars = 20 nm.

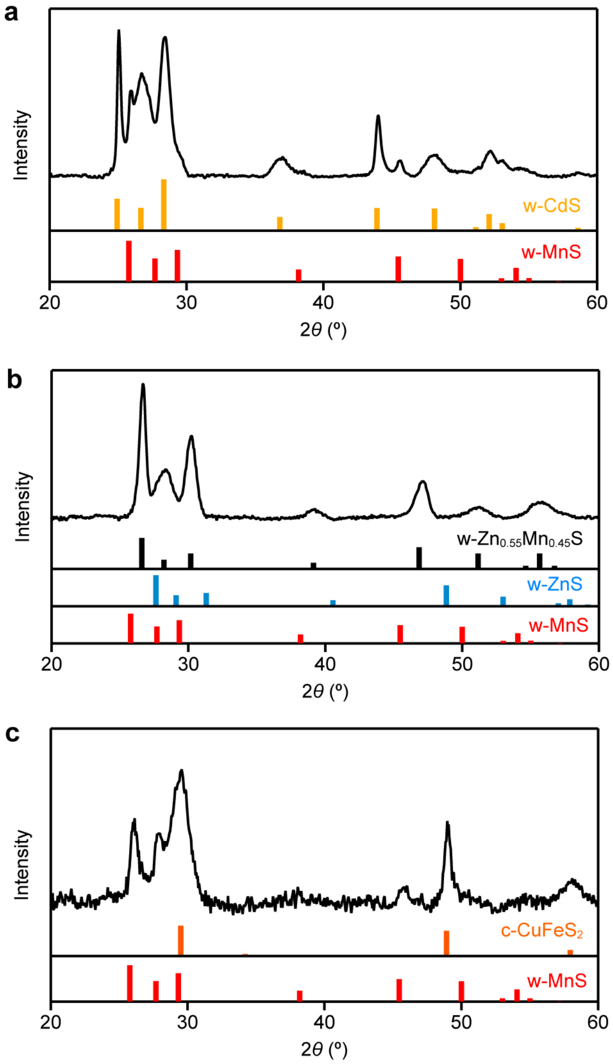

Supplementary Fig. 18 XRD analyses of HNPLs formed by waxing process of $Cu_{1.8}S$–MnS HNPLs. XRD patterns of the products generated by additional CE of $Cu_{1.8}S$–MnS HNPLs with (a) Cd^{2+}, (b) Zn^{2+}, or (c) Fe^{3+}. Reference patterns: w-CdS (yellow, ICDD-PDF 01-080-0006), w-ZnS (blue, ICDD-PDF 01-079-2201), w-$Zn_{0.55}Mn_{0.45}S$ (black, ICDD-PDF 00-011-0513), c-$CuFeS_2$ (orange, ICDD-PDF 01-075-0253).

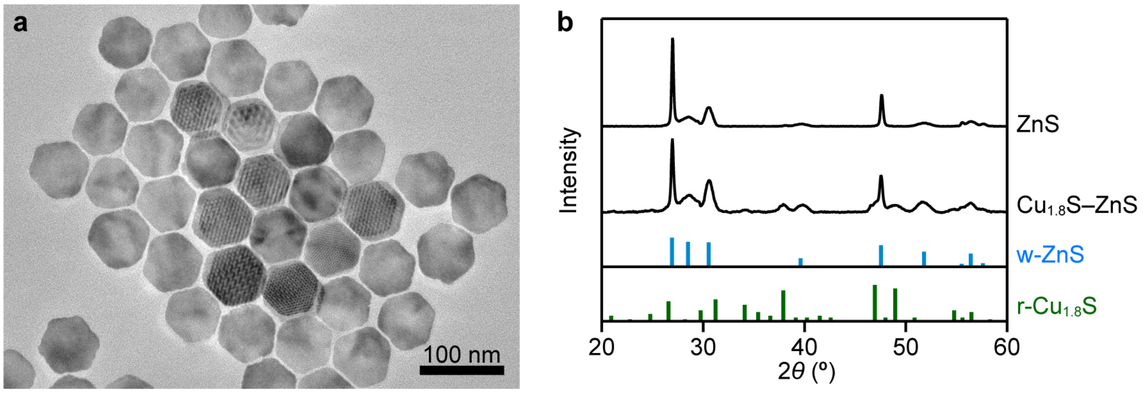

Supplementary Fig. 19 ZnS NPLs formed from additional Zn^{2+} CE of $Cu_{1.8}S$–ZnS HNPLs. (a) TEM image of ZnS NPLs obtained by additional CE of $Cu_{1.8}S$–ZnS HNPLs with Zn^{2+}. (b) XRD pattern of $Cu_{1.8}S$–ZnS HNPLs and ZnS NPLs. Reference patterns: r-$Cu_{1.8}S$ (ICDD-PDF 00-064-0278), w-ZnS (ICDD-PDF 01-079-2201). Reaction condition: [$ZnCl_2$]/[$Cu_{1.8}S$–ZnS] = 4.

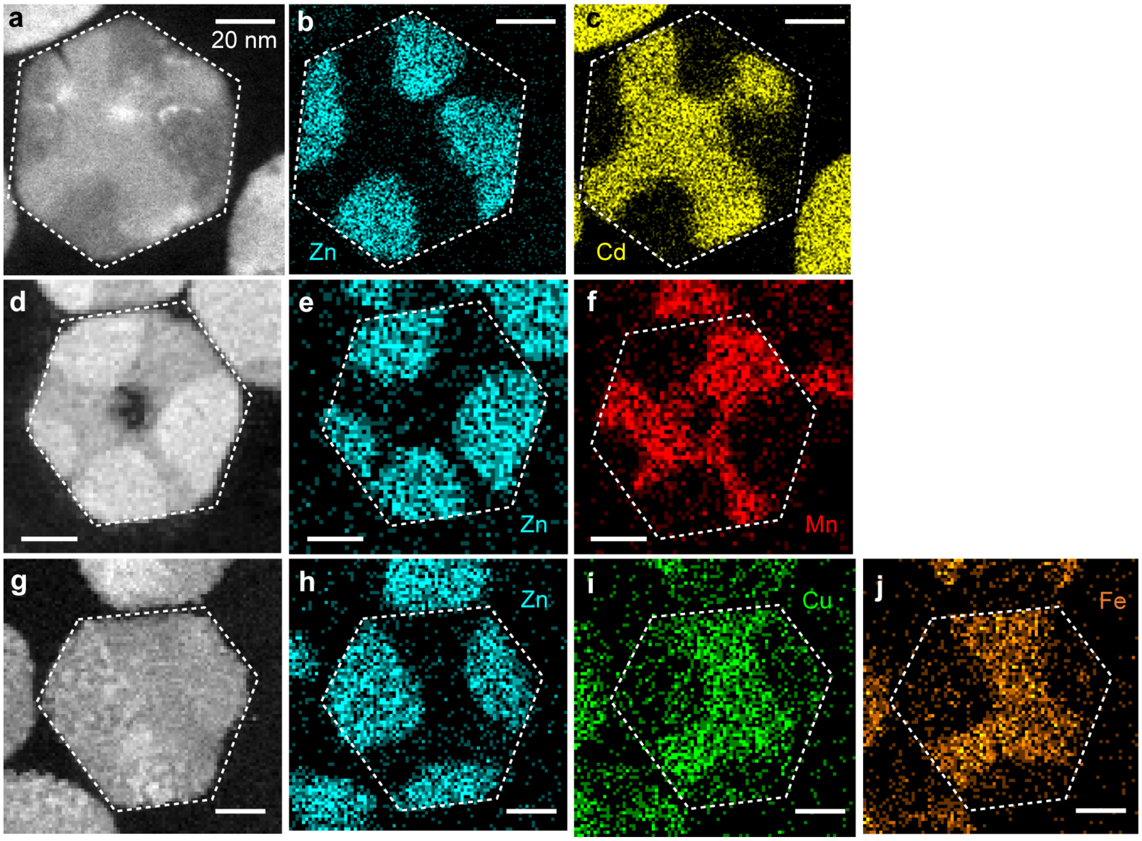

Supplementary Fig. 20 Metal cations distribution in HNPLs after waxing process of the $Cu_{1.8}S$–ZnS HNPLs. (a,d,g) HAADF-STEM images and (b,c,e,f,h,i,j) STEM–EDX maps of (a–c) ZnS–CdS HNPLs, (d–f) ZnS–MnS HNPLs and (g–j) ZnS–$CuFeS_2$ HNPLs synthesized by two-step CE. (b,e,h) Blue: Zn-K, (c) yellow: Cd-K, (f) red: Mn-K and (j) orange: Fe-K. Dotted lines outline an HNPL. Scale bars = 20 nm.

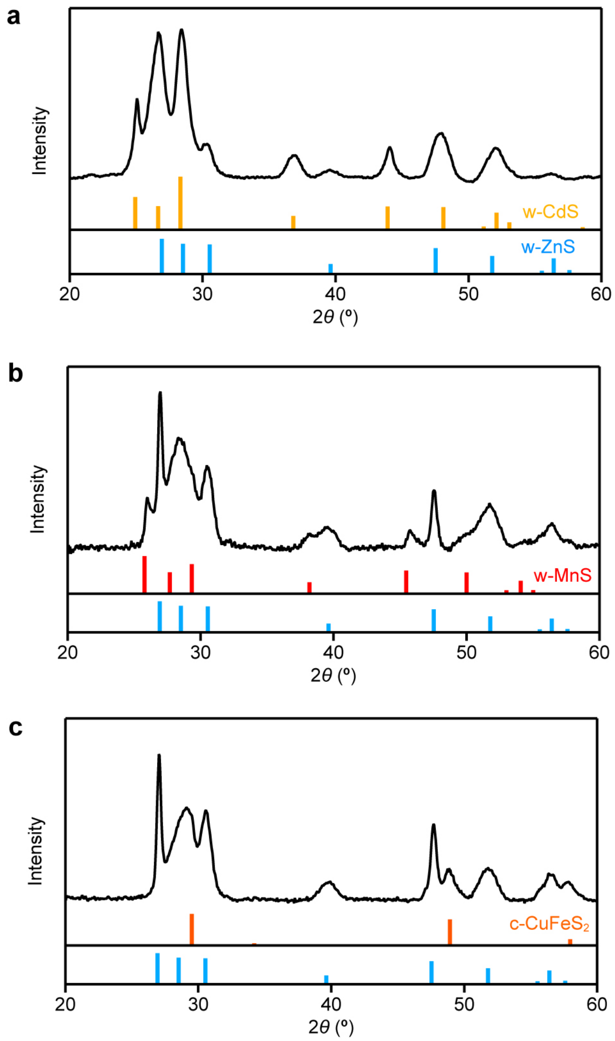

Supplementary Fig. 21 XRD analyses of HNPLs formed by waxing process of $Cu_{1.8}S$–ZnS HNPLs. XRD patterns of the products generated by additional CE of $Cu_{1.8}S$–ZnS HNPLs with (a) Cd^{2+}, (b) Mn^{2+}, or (c) Fe^{3+}. Reference patterns: w-CdS (yellow, ICDD-PDF 01-080-0006), w-ZnS (blue, ICDD-PDF 01-079-2201), c-$CuFeS_2$ (orange, ICDD-PDF 01-075-0253).

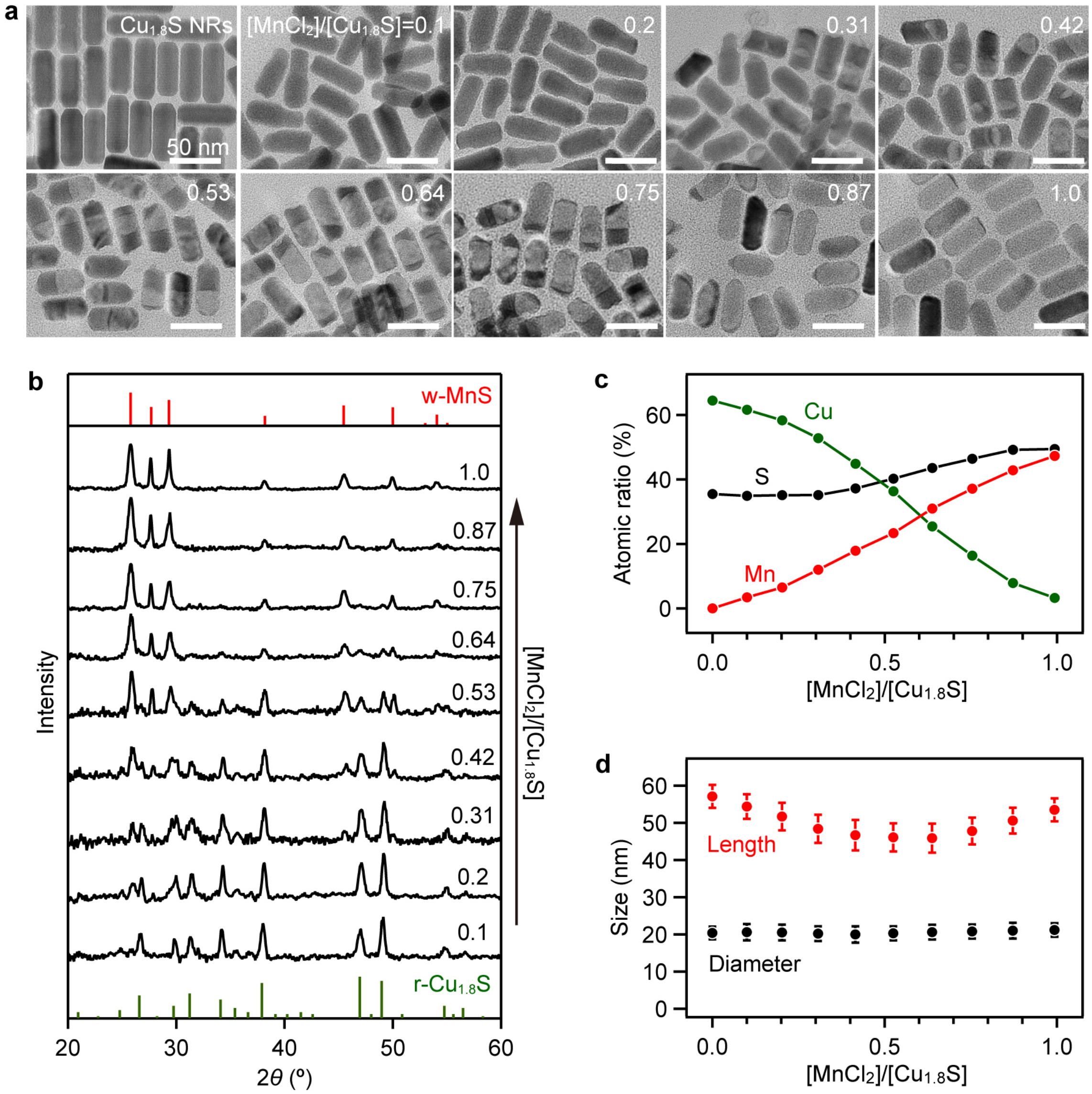

Supplementary Fig. 22 Successive Mn^{2+} CE of $Cu_{1.8}S$ NRs. (a) TEM images, (b) XRD patterns, (c) compositional changes and (d) length and diameter changes of heterostructured $Cu_{1.8}S$–MnS NRs formed by stepwise CE of $Cu_{1.8}S$ NRs with Mn^{2+}. Error bars represent standard deviations of lengths (n = 30) and diameters (n = 30). Reference XRD patterns: w-MnS (red, ICDD-PDF 01-089-4089) and r-$Cu_{1.8}S$ (green, ICDD-PDF 00-064-0278).

Supplementary Table 1 Calculated Gibbs free energy changes for CE reactions of Cu_2S with Mn^{2+}, Zn^{2+} and Cd^{2+}. [a] Here, we used the standard Gibbs energy of sphalerite-ZnS as there is no published value for the standard Gibbs energy of wurtzite-ZnS.

	ΔG_f^o (kJ mol^{-1})	E^o(V)	ΔG_{CE} (kJ mol^{-1})
Cu_2S	–86.2	+0.521	-
CdS	–156.5	–0.403	**+208.5**
ZnS	–201.3[a]	–0.762	**+233.0**
MnS	–218.4	–1.185	**+297.5**
